# Rad51 Inhibits Translocation Formation by Non-Conservative Homologous Recombination in *Saccharomyces cerevisiae*

**DOI:** 10.1371/journal.pone.0011889

**Published:** 2010-07-29

**Authors:** Glenn M. Manthey, Adam M. Bailis

**Affiliations:** Department of Molecular and Cellular Biology, Beckman Research Institute, City of Hope National Medical Center, Duarte, California, United States of America; University of Minnesota, United States of America

## Abstract

Chromosomal translocations are a primary biological response to ionizing radiation (IR) exposure, and are likely to result from the inappropriate repair of the DNA double-strand breaks (DSBs) that are created. An abundance of repetitive sequences in eukaryotic genomes provides ample opportunity for such breaks to be repaired by homologous recombination (HR) between non-allelic repeats. Interestingly, in the budding yeast, *Saccharomyces cerevisiae* the central strand exchange protein, Rad51 that is required for DSB repair by gene conversion between unlinked repeats that conserves genomic structure also suppresses translocation formation by several HR mechanisms. In particular, Rad51 suppresses translocation formation by single-strand annealing (SSA), perhaps the most efficient mechanism for translocation formation by HR in both yeast and mammalian cells. Further, the enhanced translocation formation that emerges in the absence of Rad51 displays a distinct pattern of genetic control, suggesting that this occurs by a separate mechanism. Since hypomorphic mutations in *RAD51* in mammalian cells also reduce DSB repair by conservative gene conversion and stimulate non-conservative repair by SSA, this mechanism may also operate in humans and, perhaps contribute to the genome instability that propels the development of cancer.

## Introduction

Cells are constantly challenged with an array of exogenous and endogenous agents that damage their DNA. Perhaps the most pernicious DNA lesion is the double-strand break (DSB) that can be lethal if left unrepaired [1], [2]. In eukaryotic cells, a number of repair mechanisms have evolved to address this lesion, including the homology-directed mechanisms of gene conversion (GC), break induced replication (BIR), synthesis dependent strand annealing (SDSA), and single strand annealing (SSA), while a fifth mechanism, non-homologous end joining, uses little to no homology [3], [4], [5]. Importantly, all of the homology-directed mechanisms utilize 3′ single-stranded intermediates generated by resection of the 5′ strands at the ends of DSBs [6], [7]. The single-stranded DNA binding protein, RPA, binds first at these intermediates, and is then displaced by the recombination mediator protein, Rad52 [8], [9]. Rad52 also promotes the recruitment of the strand invasion protein, Rad51, forming a nucleoprotein filament that can facilitate GC, BIR, and SDSA [10], [11], [12], [13], [14]. In contrast, Rad51 is not required for SSA, and can even be inhibitory [15], [16], [17]. This inhibitory effect is likely to be due to the ability of Rad51 to block the annealing of complementary 3′ single-strands by Rad52, a critical step in SSA [16], [18], [19].

DSB repair by SSA is an efficient mechanism of genome rearrangement that can create chromosomal deletions and translocations at high frequencies in eukaryotic species from yeast to humans [20], [21], [22], [23], [24], [25], [26], [27], [28], [29]. This is likely to be relevant to the genome instability observed following acute accidental or therapeutic exposure to IR, as numerous, widely distributed genomic DSBs are likely to stimulate SSA using repetitive genomic sequences [22], [30], [31]. Chromosomal deletions and non-reciprocal translocations by SSA may also account for variations in gene copy number that are thought to be important contributors to the development of cancer and genome evolution [32], [33].

**Figure 1:**
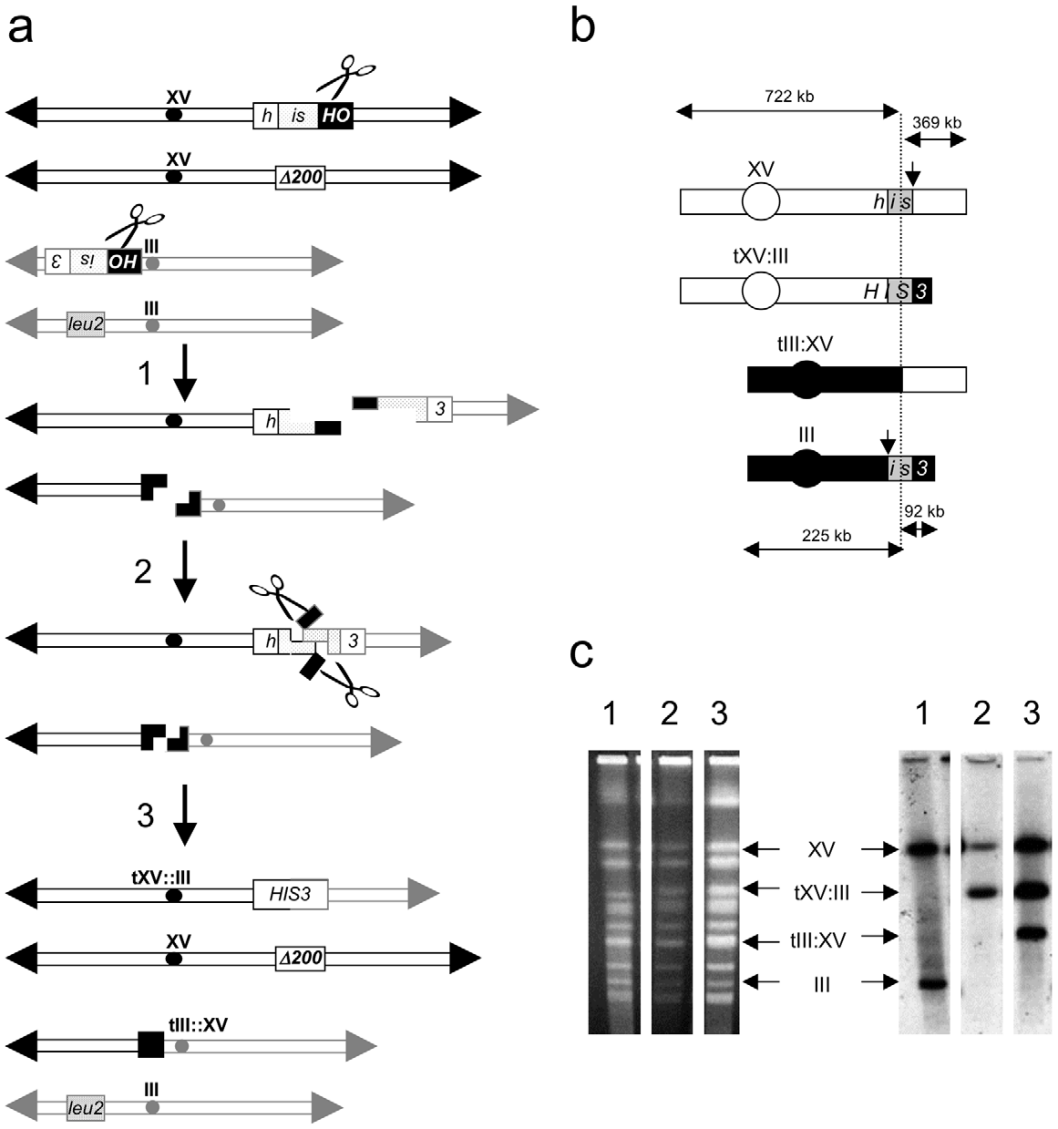
Formation of translocation chromosomes by SSA. (**a**) Translocation formation by HR between *his3* substrates after two DSBs. 1. Recombination between the *his3-*Δ*3*′ substrate at the *HIS3* locus on one copy of chromosome XV (centromeres black circles, telomeres black triangles) and the *his3-*Δ*5*′ substrate at the *LEU2* locus on one copy of chromosome III (centromeres gray circles, telomeres gray triangles) is initiated by HO endonuclease (scissors) cutting at adjacent HO cut sites in 117 bp fragments of the *y/z* junction of *MAT* ***a*** (black rectangles). The *his3-*Δ*200* allele at the *HIS3* locus on the other copy of chromosome XV lacks sufficient homology to generate an intact *HIS3* gene by recombination with either *his3-*Δ*3*′ or *his3-*Δ*5*′. Exonucleolytic processing of the ends of the broken chromosomes creates 3′ single-strands. 2. Either 60 bp or 300 bp complementary sequences in the 3′ single strands associated with *his3-*Δ*3*′ at the end of one fragment of chromosome XV, and *his3-*Δ*5*′ at the end of one fragment of chromosome III are annealed by Rad52 and Rad59, creating 3′ non-homologous tails that are the remnants of the HOcs sequences (black rectangles). Tails are removed by Rad1-Rad10 endonuclease (scissors). At the ends of the other fragments of chromosomes XV and III, the four bp sticky ends created by HO endonuclease digestion can also be annealed in a process that is independent of Rad52, Yku70 and Yku80 [27]. 3. Repair synthesis and ligation create an intact *HIS3* gene associated with the recombinant translocation chromosome tXV:III. Dnl4-independent ligation of the annealed sticky ends on the other chromosome fragments creates the recombinant translocation chromsome III:XV [27]. (**b**) Expected chromosomal products of HR between *his3* substrates. Cutting on the right side of the *his3-*Δ*3*′ homology box (gray box) at the *HIS3* locus on chromosome XV by HO endonuclease (downward facing arrow) creates 722 kb and 369 kb fragments. Cutting on the left side of the *his3-*Δ*5*′ homology box (gray box) at the *LEU2* locus on chromosome III by HO endonuclease creates 225 kb and 92 kb fragments. HR between *his3-*Δ*3*′ and *his3-*Δ*5*′ creates an intact *HIS3* coding sequence on an 814 kb tXV:III translocation chromosome. The 594 kb tIII:XV reciprocal translocation chromosome, created by a process that can utilize minimal homology between the broken ends may also appear. (**c**) Observation of recombinant translocation chromosomes by chromosome blot hybridization. Displayed on the left are chromosomes prepared from His^−^ parent and representative His^+^ recombinant strains were separated by CHEF on agarose gels, stained with ethidium bromide, and photographed. On the right are the gel-separated chromosomes that have been denatured in alkali, blotted to nylon, hybridized with a ^32^P-labeled 1.8 kb *Bam*HI genomic clone containing the *HIS3* coding sequence, and autoradiographed. Lanes: (1) His^−^ parent. (2) His^+^ recombinant carrying the tXV:III translocation chromosome. (3) His^+^ recombinant carrying the tXV:III translocation chromosome and the tIII:XV reciprocal translocation chromosome.

Recent studies of translocation formation by SSA in yeast have revealed a complex pattern of genetic control, indicating the existence of an elaborate apparatus for propagating these events. In particular, genetic interactions between the *RAD52* paralog, *RAD59*, the central mismatch repair gene, *MSH2*, and the structure-specific nuclease subunit gene, *RAD1*, suggest that SSA employs a specific apparatus for the removal of non-homologous tails formed upon annealing of complementary sequences on the 3′ single-stranded intermediates (Figure 1)[8], [27], [34], [35], [36], [37]. Removal of the tails is thought to be of critical importance because they block the repair synthesis and ligation that complete the formation of the rearranged chromosomes [37]. Loss of any of the factors that contribute to this apparatus results in severe reductions in the frequency of translocation [27], [36].

The current paper describes the effects of the loss of *RAD51* on translocation formation by SSA. By playing a potentially inhibitory role, Rad51 could comprise part of the defense against catastrophic genome rearrangement following extensive DNA damage. In support of previous studies of the genetic control of SSA, deletion of *RAD51* resulted in an increased frequency of translocation formation [15], [16], [17]. Importantly, *rad52-*329, a mutation that deletes the C-terminal domain of Rad52 that interacts with Rad51 and permits it to execute its recombination mediator function also has stimulatory effects [38], [39], [40], [41], [42]. Most strikingly, however, was the observation that both *rad51*Δ and *rad52-329* suppressed the requirement for Srs2, Rad1 and Rad59, factors that are otherwise necessary for translocation formation by SSA, indicating that a distinct and more efficient mechanism of translocation formation can replace the primary SSA mechanism. This may have important implications with respect to the development of cancer in humans as mutations in *RAD51*, and *BRCA2*, which encodes a tumor suppressor protein with a recombination mediator function, confer similar increases in SSA [17], [23], [43], [44], [45], [46], [47]. Rad51 may, therefore play a critical, evolutionarily conserved role in the maintenance of genome stability by moderating the levels of DSB repair by potent non-conservative mechanisms.

## Results

### Rad51 is required for conservative HR between sequences on different chromosomes but inhibits non-conservative HR that leads to translocations

We have used several assays to study HR between duplicate sequences on non-homologous chromosomes in budding yeast. Translocation formation by HR between 3′ and 5′ truncated alleles of the *HIS3* gene that share 60 bp or 300 bp of identical sequence, and are located at the *HIS3* locus on one copy of chromosome XV and the *LEU2* locus on one copy of chromosome III has figured prominently in recent work [27], [36], [48], [49]. The assay is performed three ways that differ in the manner by which recombination is initiated: T0, where recombination occurs spontaneously, T1, where recombination is stimulated by an HO endonuclease-mediated DSB adjacent to one of the *his3* substrates, and, T2, where recombination is stimulated by DSBs adjacent to both substrates (Figure 1)[27]. The efficiency that a functional *HIS3* gene and tXV::III translocation chromosome are formed in wild type diploids depends on the mode of inititation, with T0 occurring at a rate of 6.0×10^−9^, T1 at a frequency of 1.4×10^−5^, and T2 at a frequency of 2.2×10^−2^ (Table 1)[27]. While the structure of the tXV:III translocation chromosome appears identical in nearly all recombinants, the structures of the occasional reciprocal tIII:XV translocations are distinct in T0, T1 and T2, as are the karyotypes of the recombinants, which can include different numbers of chromosomes carrying intact substrates (G. Manthey and A. Bailis – unpublished observations). Further, the genetic control of these processes is distinct as mutations in several DNA repair and HR genes exert different effects on T0, T1 and T2 [27], [36], [49]. The accumulated evidence, therefore, strongly supports the conclusion that T0, T1 and T2 proceed by distinct mechanisms.

**Table 1:** Spontaneous or DSB-stimulated interchromosomal recombination and plating efficiencies in wild type and homozygous mutant diploid strains.

Genotype^a^	EGC^b^	T0^c^	T1^d^	T2^e^	PE^f^	
					Pre	Post
Wild type	1.1×10^−3^	6.0×10^−9^	1.4×10^−5^	2.2×10^−2^	2.1×10^−1^	1.6×10^−1^
	(0.6, 1.6)	(4.4, 7.6)	(0.8, 3.2)	(1.4, 3.1)	(1.4, 4.4)	(1.1, 3.6)
*rad51*Δ*/rad51*Δ	7.4×10^−7^	1.1×10^−7^	2.5×10^−5^	6.0×10^−2^	ND	ND
	[−1487]	[+18.3]	[+1.8]	[+2.7]		
	(4.0, 11.8)	(0.9, 1.4)	(1.8, 3.3)	(3.8, 12)		
*rad52*Δ*/rad52*Δ	3.6×10^−7^	<8.4×10^−10^	2.3×10^−7^	3.0×10^−3^	1.8×10^−1^	4.2×10^−2^
	[−3056]	[>−7.1]	[−61]	[−7.3]	[−1.2]	[−3.8]
	(1.6, 5.6)		(1.2, 7.2)	(1.3, 4.3)	(1.3, 3.5)	(3.1, 7.0)
*rad52-329/rad52-329*	3.2×10^−7^	ND	3.7×10^−5^	2.0×10^−2^	2.9×10^−1^	1.9×10^−1^
	[−3438]		[+2.6]	[−1.1]	[+1.4]	[+1.2]
	(2.7, 4.2)		(2.4, 5.8)	(1.4, 3.1)	(2.5, 3.9)	1.7, 3.5)

a All diploid strains were homozygous for *MATa::LEU2* such that no cutting by HO endonuclease occurred at the *MAT* locus, except for T0 strains where HO endonuclease is not expressed.

b Frequencies of ectopic gene conversion (EGC) in diploid cells between *sam1-*Δ*Bgl*II at the *SAM1* locus on one copy of chromosome XII and *sam1-*Δ*Sal*I at the *HIS3* locus on one copy of chromosome XV were determined following a HO endonuclease-mediated break in *sam1-*Δ*Bgl*II as described in the Materials and Methods. Median frequencies were determined from a minimum of 10 independent cultures for each strain. Fold differences from the median frequency observed with wild type strains are indicated in brackets with fold increases preceded by a “plus” and fold decreases preceded by a “minus“. The 95% confidence intervals are indicated in parentheses. Frequencies in wild type and *rad52*Δ*/rad52*Δ homozygotes described previously [49].

c Rates of translocation formation in diploid cells by spontaneous HR (T0) between a 300 bp segment of the *HIS3* coding sequence shared by the *his3-*Δ*5*′ substrate at the *LEU2* locus on one copy of chromosome V and a *his3-*Δ*3*′ substrate at the *HIS3* locus on one copy of chromosome XV were determined using the method of the median from a minimum of 10 independent trials as described in the Materials and Methods. Fold differences from wild type and 95% confidence intervals are displayed as described above. ND  =  not determined. These results were published previously [27].

d Frequencies of translocation formation in diploid cells by HR between a 300 bp segment of the *HIS3* coding sequence shared by the *his3-*Δ*5*′ substrate at the *LEU2* locus on one copy of chromosome III and a *his3-*Δ*3*′ substrate at the *HIS3* locus on one copy of chromosome XV were determined following a HO endonuclease-mediated break adjacent to the *his3-*Δ*5*′ substrate (T1) as described in the Materials and Methods. Median frequencies, fold differences from wild type and 95% confidence intervals are displayed as described above.

e Frequencies of translocation formation in diploid cells by HR between *his3-*Δ*5*′ and *his3-*Δ*3*′ following HO endonuclease-mediated breaks adjacent to both substrates (T2) were determined as described in the Materials and Methods. Median frequencies, fold differences from wild type and 95% confidence intervals are displayed as described above.

f Plating efficiencies (PE) “Pre” and “Post” HO endonuclease cutting at *his3-*Δ*5*′ and *his3-*Δ*3*′ were determined as described in the Materials and Methods, and previously. Median frequencies, fold differences from wild type and 95% confidence intervals are displayed as described above.

In addition to assays for non-conservative HR between chromosomes, we have utilized an assay that measures the repair of a HO-stimulated DSB by ectopic gene conversion (EGC) between duplicate sequences on non-homologous chromosomes in diploids [49]. A DSB is generated in the *sam1-*Δ*BglII-HOcs* allele at the *SAM1* locus on one copy of chromosome XII, and repaired by gene conversion with the *sam1-*Δ*SalI* allele, located at the *HIS3* locus on one copy of chromosome XV. In wild-type diploids, EGC creates a functional *SAM1* gene at a frequency of 1.1×10^−3^ (Table 1)[49]. Translocation formation by reciprocal recombination between the *sam1* alleles cannot be observed in this assay because it would produce dicentric chromosomes due to the opposite orientation of the *sam1* alleles relative to their centromeres.

We had previously shown that the frequency of EGC is reduced over 3,000-fold in *rad52*Δ*/rad52*Δ homozygotes, consistent with these events being highly dependent on Rad52 (Table 1)[49]. In the current study, loss of Rad51 led to a nearly equivalent (p = 0.20), 1,500-fold decreased frequency in *rad51*Δ*/rad51*Δ homozygotes, consistent with EGC proceeding by a strand exchange-mediated mechanism [12]. Further, in *rad52-329/rad52-329* homozygotes, where C-terminally deleted Rad52 is defective in its interaction with Rad51, EGC is also reduced approximately 3,000-fold, consistent with results with other systems [38]. This suggests that the recombination mediator function of Rad52 is required for Rad51 to exert its effect, and it is this function of Rad52 that is required for EGC. Together, these data suggest that the repair of a DSB by conservative gene conversion using homologous sequences on different chromosomes requires the involvement of both Rad51 and Rad52, and that Rad51 and Rad52 must interact.

**Figure 2 pone-0011889-g002:**
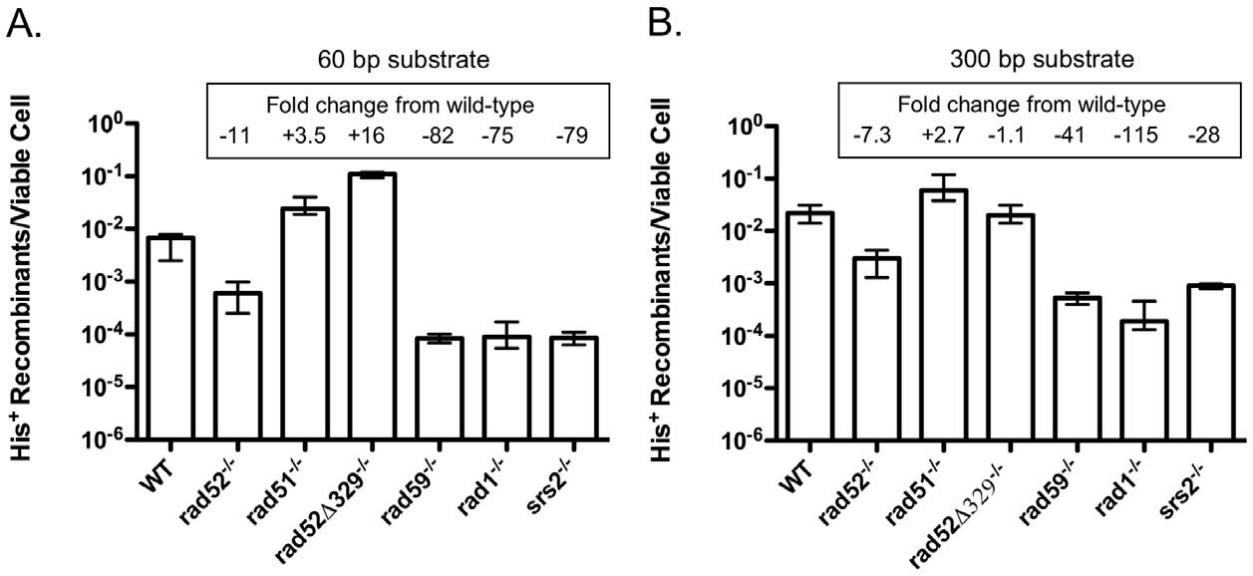
Frequencies of translocation following DSB formation by HO endonuclease adjacent to both *his3* substrates in homozygous wild-type and DNA repair defective mutant diploids. (**a**) Frequencies of His^+^ colony formation by HR between 60 bp substrates. Frequencies of His^+^ colony formation from a minimum of 10 trials with each strain were determined as discussed in the Materials and Methods. Median frequencies are depicted. Error bars represent 95% confidence intervals determined as described in the Materials and Methods. Fold decreases from wild-type depicted by quantities following a “minus” sign. Fold increases from wild-type depicted by quantities following a “plus” sign. Actual frequencies presented in Table S1. (**b**) Frequencies of His^+^ colony formation by HR between 300 bp substrates.

Our studies of the formation of chromosomal translocations by T0, T1 and T2 have demonstrated that distinct mechanisms are utiIized [27], [36], [49]. But, like EGC, all translocation formation displays some degree of dependence on Rad52, as it is reduced by seven- to 61-fold in *rad52*Δ*/rad52*Δ homozygotes (Table 1, Figure 2). In contrast, while EGC is acutely dependent on Rad51, T0, T1 and T2 are inhibited by Rad51, as translocation formation is increased from approximately two- to 18-fold in *rad51*Δ*/rad51*Δ homozygotes. Abrogating the interaction between Rad51 and Rad52 in *rad52-329/rad52-329* homozygotes has similar effects, indicating that translocation formation does not require the mediator function of Rad52, and suggesting that Rad51 cannot inhibit translocation formation if it cannot associate efficiently with DSBs. The different effects of the mutant alleles in these assays is unlikely to be due to differences in the ability to survive DSBs as plating efficiencies are substantially similar (Table 1). Considering the gene conversion and translocation data together suggests that the collaboration between Rad51 and Rad52 that potentiates DSB repair by conservative inter-chromosomal HR also limits repair by non-conservative inter-chromosomal HR.

### The genetic control of T2 is consistent with SSA

T2 is the most efficient mechanism of translocation formation by HR in both yeast and mammalian cells, and may account for the high frequency of translocations observed in acutely irradiated yeast cells (Table 1)[27], [29], [30]. We have shown previously that T2 displays a pattern of genetic control consistent with SSA, including a considerable dependence on *RAD1* and *RAD59* that work together in supporting the removal of non-homologous tails [27], [49]. Consistent with our previous analyses, the *rad1*Δ*/rad1*Δ and *rad59*Δ*/rad59*Δ homozygotes both displayed strongly diminished frequencies of translocation, 75- and 41-fold reduced with the 60 bp substrates, and 115- and 82-fold reduced with the 300 bp substrates (Figure 2). Similarly, Haber and colleagues had previously shown that DSB-stimulated deletion formation by SSA is also dependent on *SRS2*, which encodes a helicase that facilitates the removal of Rad51 from the 3′ single strands that form at DSBs [35], [50], [51], [52]. Consistent with their findings T2 was found to be dependent on Srs2, as *srs2*Δ*/srs2*Δ homozygotes exhibited 79- and 24-fold reduced frequencies of translocation with the 60 bp and 300 bp substrates, respectively.

**Figure 3 pone-0011889-g003:**
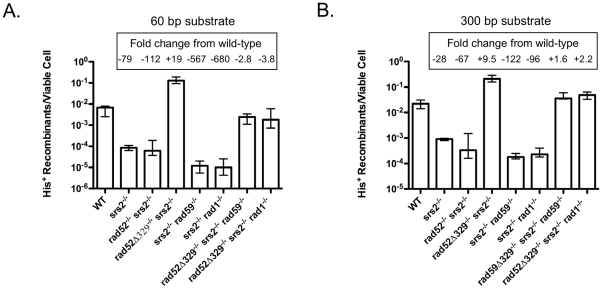
Frequencies of translocation following DSB formation by HO endonuclease adjacent to both *his3* substrates in wild-type, and single, double and triple DNA repair defective homozygotes. (**a**) Frequencies of His^+^ colony formation by HR between 60 bp substrates. Median frequencies and confidence intervals were determined as described in the legend to Figure 2 and in the Materials and Methods. Fold decreases from wild-type depicted by quantities following a “minus” sign. Fold increases from wild-type depicted by quantities following a “plus” sign. Actual frequencies presented in Table S1. (**b**) Frequencies of His^+^ colony formation by HR between 300 bp substrates.

Epistasis analysis was used previously to determine that *RAD1* and *RAD59* work together to control the removal of non-homologous tails during T2, as *rad1* and *rad59* alleles were observed to confer mutually suppressive effects [27], [49]. Accordingly, we examined the epistasis relationships between *srs2*Δ, and both *rad1*Δ and *rad59*Δ. Interestingly, with the 60 bp substrates *srs2*Δ displayed a synergistic interaction with both *rad1*Δ and *rad59*Δ as translocation frequencies were reduced 680-fold in the *srs2*Δ*/srs2*Δ *rad1*Δ*/rad1*Δ double homozygote and 567-fold in the *srs2*Δ*/srs2*Δ *rad59*Δ*/rad59*Δ double homozygote, well below the frequencies observed in any of the single homozygotes (Figures 2 and 3). A similar relationship was observed for *srs2*Δ and *rad59*Δ when the 300 bp substrates were used as the 122-fold reduced translocation frequency in the *srs2*Δ*/srs2*Δ *rad59*Δ*/rad59*Δ double homozygote was significantly lower than the frequencies observed in the *srs2*Δ*/srs2*Δ and *rad59*Δ*/rad59*Δ homozygotes (p<0.0001). However, *rad1*Δ appeared to be epistatic to *srs2*Δ when the 300 bp substrates were used as the 96-fold reduced frequency of translocation in the *srs2*Δ*/srs2*Δ *rad1*Δ*/rad1*Δ double homozygote was not significantly different from the frequency in the *rad1*Δ*/rad1*Δ homozygote (p = 0.36), suggesting that Rad1 and Srs2 may work together with the longer substates. Overall, the pattern of interaction is most consistent with Srs2 playing a distinct role in T2 from those played by Rad1 and Rad59, although these interactions are affected by substrate length.

The *srs2*Δ allele also displayed interesting epistasis interactions with *rad52*Δ, as in the *rad52*Δ*/rad52*Δ *srs2*Δ*/srs2*Δ double homozygote the 112-fold decreased translocation frequency with the 60 bp substrates was not significantly different from the frequency in the *srs2*Δ*/srs2*Δ homozygote (p = 0.37) (Figures 2 and 3), while the 67-fold decreased frequency with the 300 bp substrates was significantly lower than the frequencies in either the *rad52*Δ*/rad52*Δ or *srs2*Δ*/srs2*Δ homozygotes (p<0.011)(Figures 2 and 3). This suggests that Srs2 and Rad52 operate together during translocation formation by SSA with the 60 bp substrates, but that they may function separately with the 300 bp substrates. The collective pattern of T2 epistasis was consistent with Srs2 working with Rad52, and separate from Rad1 and Rad59 for translocation formation with the 60 bp substrates, and with Rad1, but separate from Rad52 and Rad59 with the 300 bp substrates.

### Association between Rad51 and Rad52 mandates the utilization of Rad1, Rad59 and Srs2 in the generation of translocations by SSA

**Figure 4:**
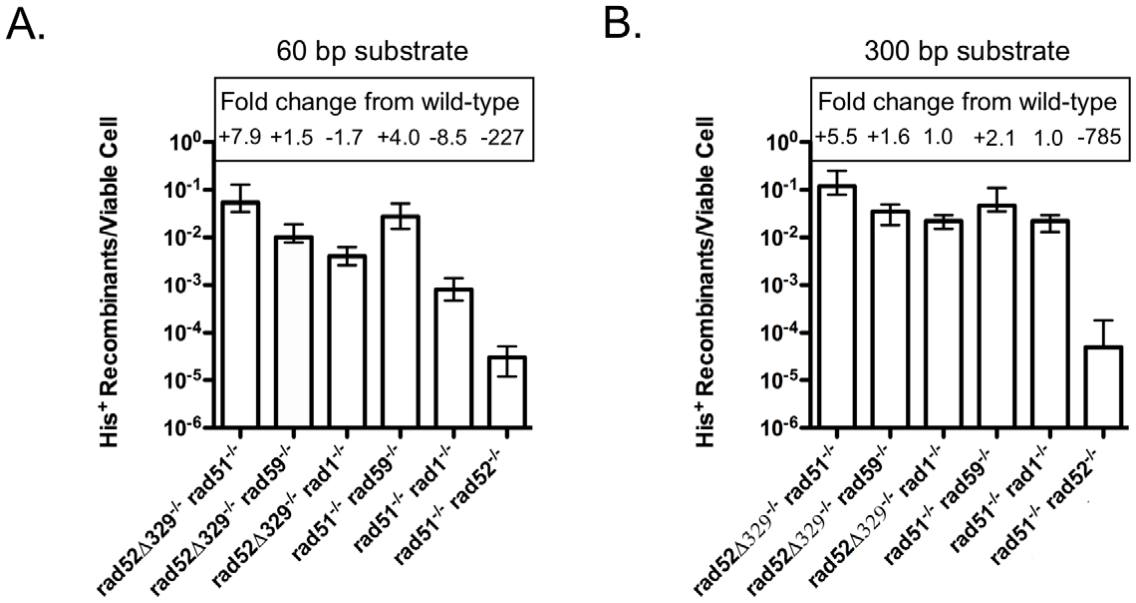
Frequencies of translocation following DSB formation by HO endonuclease adjacent to both *his3* substrates in DNA repair defective double homozygotes. (**a**) Frequencies of His^+^ colony formation by HR between 60 bp substrates. Median frequencies and confidence intervals were determined as described in the legend to Figure 2 and in the Materials and Methods. Fold decreases from wild-type depicted by quantities following a “minus” sign. Fold increases from wild-type depicted by quantities following a “plus” sign. Actual frequencies presented in Table S1. (**b**) Frequencies of His^+^ colony formation by HR between 300 bp substrates.

Observing the stimulation of T2 in the *rad51*Δ*/rad51*Δ and *rad52-329/rad52-329* homozygotes suggests that the loss of Rad51, and the truncation of Rad52 such that it cannot interact with Rad51 can both stimulate translocation formation by SSA (Table 1, Figure 2). However, the frequencies of translocation were significantly different with both the 60 bp substrates, where the 16-fold increased frequency in the *rad52-329/rad52-329* homozygote exceeded the 3.5-fold increased frequency in the *rad51*Δ*/rad51*Δ homozygote (p = 0.0001), and the 300 bp substrates, where the 2.7-fold increased frequency in the *rad51*Δ*/rad51*Δ homozygote was greater than the essentially wild type frequency in the *rad52-329/rad52-329* homozygote (p = 0.0018)(Figure 4). These results indicate that *rad51*Δ and *rad52-329* have non-equivalent effects on T2, which were studied further through epistasis analysis. The 7.9-fold increased frequency of T2 with the 60 bp substrates in the *rad51*Δ*/rad51*Δ *rad52-329/rad52-329* double homozygote was intermediate to and significantly different from the frequencies in both the *rad51*Δ*/rad51*Δ (p = 0.0062) and *rad52-329/rad52-329* (p = 0.0043) homozygotes, indicating that *rad51*Δ partially suppressed the effect of *rad52-329*. The 5.5-fold increased frequency with the 300 bp substrates in the *rad51*Δ*/rad51*Δ *rad52-329/rad52-329* double homozygote was significantly greater than those observed in either the *rad51*Δ*/rad51*Δ (p = 0.0009) or *rad52-329/rad52-329* (p = 0.0001) homozygotes, indicating that *rad51*Δ and *rad52-329* had a mildly synergistic effect. This pattern of genetic interaction is most consistent with the *rad51*Δ and *rad52-329* alleles increasing T2 by similar mechanisms and suggests that their effects, while different in degree may be essentially interchangeable genetically.

The loss of Rad51 in the *rad51*Δ*/rad51*Δ homozygote, and the inability of Rad52 to associate with Rad51 in the *rad52-329/rad52-329* homozygote would both be expected to block formation of Rad51 nucleoprotein filaments at DSBs [40], [42], [53]. The increased frequencies of T2 in these mutants suggest that loss of filament formation might alter the manner in which the factors that mediate SSA associate with the recombination substrates, so we examined the genetic control of T2 in *rad51*Δ*/rad51*Δ and *rad52-329/rad52-329* homozygotes. Since Srs2 has been demonstrated to remove Rad51 from 3′ single-stranded DNA *in vitro*, we examined the effect of losing Srs2 in the context of a *rad52-329/rad52-329* homozygote, where Rad51 filament formation should be at a minimum [51], [52], [54], [55]. The frequency of translocation formation with 60 bp substrates in the *rad52-329/rad52-329 srs2*Δ*/srs2*Δ double homozygote was increased 19-fold over wild type, which was not significantly different from the 16-fold increase observed in the *rad52-329/rad52-329* homozygote (p = 0.078), indicating that the 79-fold reduction in T2 conferred by *srs2*Δ is completely suppressed, and that *rad52-329* is epistatic to *srs2*Δ (Figures 2 and 3). Similarly, with the 300 bp substrates T2 was increased 9.5-fold in the *rad52-329/rad52-329 srs2*Δ*/srs2*Δ double homozygote, indicating complete suppression of the 24-fold reduction in T2 by *srs2*Δ. These results are consistent with Srs2 promoting translocation formation by SSA by acting in the removal of Rad51 from the 3′ single-stranded DNA that forms at DSBs, and that this function is not required when Rad52 cannot facilitate nucleoprotein filament formation by Rad51.

Like Srs2, Rad59 has previously been shown to be an important factor in SSA, and is critically important for T2 (Figure 2)[27], [35], [49], [56]. In addition to displaying an annealing activity of its own, Rad59 partially mitigates the inhibitory effect of Rad51 nucleoprotein filaments on the annealing of complementary single-stranded DNA molecules by Rad52 *in vitro*, consistent with Rad59 playing a role in the annealing step of SSA (Figure 1)[18], [56], [57], [58]. Further, *rad59*Δ exhibits epistasis relationships with both *msh2*Δ and *rad1*Δ that are consistent with Rad59 playing a critical role in the removal of non-homologous tails [27], [36], [49]. Together, these data indicate that Rad59 plays multiple roles in T2. Intriguingly, both *rad51*Δ and *rad52-329* suppressed the effects of losing Rad59, as translocation frequencies with the 60 bp and 300 bp substrates that were 82- and 41-fold decreased in the *rad59*Δ*/rad59*Δ homozygotes were 4.0- and 2.1-fold increased in the *rad51*Δ*/rad51*Δ *rad59*Δ*/rad59*Δ double homozygotes and 1.5- and 1.6-fold increased in the *rad52-329/rad52-329 rad59*Δ*/rad59*Δ double homozygotes (Figures 2 and 4). These data indicate that Rad59 is not required for T2 in the absence of Rad51 nucleoprotein filament formation, but also suggest that it is the presence of filaments that specifies the requirement for Rad59 in T2.

The requirement for the heterodimeric endonuclease Rad1-Rad10 in SSA is well established, including for T2 (Figure 2)[27], [36], [49], [59], [60]. Rad1-Rad10 has been shown to cleave a variety of DNA structures associated with DNA repair and HR *in vitro*, including structures like the 3′ non-homologous tails postulated to form during T2 when complementary sequences in the 3′ single strands at the ends of the broken chromosomes are annealed (Figure 1)[61], [62], [63], [64]. Rad1-Rad10 also functions in the context of Rad51-dependent HR when non-homologous tails on DSBs must be removed to facilitate completion of the recombination event [37], [59], [60], [65]. Given the importance of Rad1-Rad10 for a variety of DSB-stimulated HR events it was surprising to observe that the *rad51*Δ and *rad52-329* mutations suppressed the 75- and 115-fold reduced frequencies of T2 with the 60 bp and 300 bp substrates, as the translocation frequencies in the *rad51*Δ*/rad51*Δ *rad1*Δ*/rad1*Δ double homozygotes were wild-type, or reduced only 8.5-fold, and the frequencies in the *rad52-329/rad52-329 rad1*Δ*/rad1*Δ double homozygotes were wild-type (Figures 2 and 4). These results indicate that Rad1-Rad10 plays a minimal role in the absence of Rad51 nucleoprotein filament formation. Further, the data suggest that the presence of filaments specifies how 3′ non-homologous tails are processed during translocation formation by SSA.

The pattern of robust suppression of the requirement for *SRS2*, *RAD59* and *RAD1* by the *rad51*Δ and *rad52-329* mutations is consistent with the loss of Rad51 filament formation permitting T2 to proceed without elements of the apparatus generally considered to be necessary for annealing and non-homologous tail removal (Figure 1)[35], [37], [59], [60], [65] [27], [36], [49]. Synergistically reduced levels of T2 in the *srs2*Δ*/srs2*Δ *rad1*Δ*/rad1*Δ and *srs2*Δ*/srs2*Δ *rad59*Δ*/rad59*Δ double homozygotes relative to the *srs2*Δ*/srs2*Δ, *rad1*Δ*/rad1*Δ and *rad59*Δ*/rad59*Δ single homozygotes support the notion that multiple aspects of the T2 mechanism are affected by these mutations (Figures 2 and 3). Consistent with loss of filament formation simultaneously affecting both annealing and tail removal, we found that *rad52-329* suppressed the synergistically reduced frequencies of T2 in the double homozygotes. This was revealed by the 567- and 122-fold reduced frequencies with 60 bp and 300 bp subunits in the *srs2*Δ*/srs2*Δ *rad59*Δ*/rad59*Δ double homozygotes becoming 2.8-fold reduced and 1.6-fold increased frequencies in the *rad52-329/rad52-329 srs2*Δ*/srs2*Δ *rad59*Δ*/rad59*Δ triple homozygotes, and the 680- and 96-fold reduced frequencies in the *srs2*Δ*/srs2*Δ *rad1*Δ*/rad1*Δ double homozygotes becoming 3.9-fold reduced and 2.2-fold increased frequencies in the *rad52-329/rad52-329 srs2*Δ*/srs2*Δ *rad1*Δ*/rad1*Δ triple homozygotes (Figure 3). This extraordinary degree of suppression indicates that T2 can dispense with much of the canonical SSA apparatus in the absence of Rad51 filament formation, but also strongly suggests that the presence of filaments mandates the use of these same factors for translocation formation by SSA.

### Rad52 is essential for translocation formation by T2 in the absence of Rad51

Rad52 is the central HR protein in budding yeast, playing a demonstrated role in almost all Rad51-dependent and –independent HR [3], [4], [5], [66]. The Rad51-independent function of Rad52 is widely understood to be related to its ability to anneal complementary single strands of DNA [19], [67], [68]. During T2, the formation of complementary single stranded DNA sequences at the ends of the broken chromosomes should greatly favor their interaction by Rad52-dependent annealing, particularly when there are no Rad51 filaments present to inhibit it (Figure 1)[18]. Consistent with this hypothesis, the *rad51*Δ*/rad51*Δ *rad52*Δ*/rad52*Δ double homozygotes displayed 227- and 785-fold reduced frequencies of translocation with the 60 bp and 300 bp substrates, which are 21- and 108-fold lower than those observed in the *rad52*Δ*/rad52*Δ homozygotes (Figures 2 and 4). These results indicate that Rad52 plays a crucial, and, perhaps predominant role in T2 in the absence of Rad51 filament formation. Further, it also suggests that Rad51 influences translocation formation in the absence of Rad52, perhaps through interactions that are independent of nucleoprotein filament formation [69].

## Discussion

Eukaryotes possess a characteristic abundance of short, repetitive sequences scattered throughout their genomes [70], [71], [72], [73], [74]. As a consequence, catastrophic levels of DNA damage, such as those created by acute exposure to IR, or DNA damaging chemicals have the potential to generate genome rearrangements by interactions between homologous repetitive sequences on the same or different chromosomes [30], [75], [76], [77], [78]. For instance, exposing diploid budding yeast cells to levels of IR sufficient to cause hundreds of DSBs per genome, with several in or near the hundreds of short, delta repeats strewn throughout the genome results in the formation of an abundance of chromosomal translocations by HR between unlinked repeats [30]. This vigorous biological response to acute levels of DNA damage suggests that non-conservative HR between repetitive genomic sequences can be a potent mechanism for genome rearrangement with important implications regarding the advent of cancer and eukaryotic genome evolution [32], [79], [80].

While HR can be an important mechanism of genomic change in response to DNA damage, it is also an important mechanism for damage tolerance. For instance, in budding yeast resistance to IR is primarily dependent on HR [2], [81], [82]. However, this resistance is most likely obtained through conservative HR between homologous sequences on sister chromatids or allelic sequences on homologous chromosomes, since the majority of survivors of even the most acute exposure display normal karyotypes [30]. Further, the genetic control of radiation survivorship resembles that of conservative HR events, such as gene conversion more than it does non-conservative HR, such as SSA. For example, the repair of a HO catalyzed DSB by gene conversion and IR resistance are both greatly dependent on Rad51, while DSB-stimulated deletion and translocation formation by SSA are not (Table 1)[12], [16], [27], [83]. Thus, DSBs can be repaired by separate conservative and non-conservative mechanisms of HR, suggesting that the maintenance of genome stability following DNA damage may require that the cell promotes one while it inhibits the other.

The data presented in this paper suggest that the interaction between Rad51 and Rad52 that facilitates Rad51 nucleoprotein filament formation may be crucial to the maintenance of genome stability by facilitating conservative HR while opposing non-conservative HR. Strikingly, whether translocation formation by HR was initiated spontaneously, or by one, or two DSBs, either the *rad51*Δ allele that results in the total loss of Rad51, or the *rad52-329* allele that results in the loss of the interaction between Rad51 and Rad52, stimulates translocation formation (Table 1 and Figure 2). This suggests that filament formation can inhibit a mechanistically diverse set of non-conservative HR events. Further, it suggests that releasing the attenuating effect of filament formation would have a broadly destabilizing effect on the genome.

Previously, the attenuating effect of *RAD51* on SSA, such as its effect on T2 was interpreted as reflecting a competition between separate apparatus for strand invasion-dependent and -independent events (Table 1 and Figure 2)[16], [17], [25]. While competition between these processes may contribute to the balance between conservative and non-conservative HR, the genetic data described in this paper clearly suggested that it is not simply the presence or absence of filaments at DSBs that steers them toward conservative and away from non-conservative mechanisms of repair. Epistasis analysis clearly suggested that Rad51 filament formation specifies the necessity for the canonical SSA machinery encoded by *SRS2*, *RAD1* and *RAD59,* as these genes were largely dispensable for T2 in *rad51*Δ*/rad51*Δ and *rad52-329/rad52-329* homozygotes (Figures 3 and 4). This suggests that in wild type cells, filaments are present at DSBs regardless of whether they are engaged in strand invasion-dependent or –independent repair. Further, this suggests the existence of a novel, and highly efficient alternative mechanism of non-conservative HR that resembles SSA but displays distinct genetic control.

Previous investigation into the interaction between Srs2 and Rad51 during the formation of chromosomal deletions by SSA suggested that loss of Srs2 does not inhibit SSA, but, instead inhibits recovery from a Rad51-dependent DNA damage checkpoint [84]. Therefore, the reduced frequencies of translocation formation observed in *srs2*Δ*/srs2*Δ homozygotes (Figure 2), as well as the epistasis interactions between *srs2*Δ, and *rad1*Δ. *rad52*Δ, *rad52-329* and *rad59*Δ (Figure 3) could be due to effects on checkpoint recovery. This possibility was explored by determining the plating efficiency before and after DSB formation in *srs2*Δ*/srs2*Δ homozygotes, and *srs2*Δ*/srs2*Δ *rad1*Δ*/rad1*Δ and *srs2*Δ*/srs2*Δ *rad59*Δ*/rad59*Δ double homozygotes (Table S3). Because plating efficiencies in the mutants were not reduced from wild type levels, changes in checkpoint recovery were not indicated. This suggests that the effects of *srs2*Δ on translocation frequency were unlikely to be due to changes in checkpoint recovery. Failure to observe altered checkpoint recovery in our experiments may be due to our use of diploid strains, where broken chromosomes have intact homologs with which to pair and attenuate the checkpoint response. Alternatively, they may be due to the fact that the DSBs occur very close to the translocation substrates, obviating the necessity to create extensive lengths of single-stranded DNA before complementary sequences are revealed.

The T2 that proceeds in the absence of Rad51, while independent of the bulk of the canonical SSA machinery, is markedly more dependent on Rad52, as translocation frequencies are 21- and 108-fold lower with the 60 bp and 300 bp substrates in the *rad51*Δ*/rad51*Δ *rad52*Δ*/rad52*Δ double homozygotes than in the *rad52*Δ*/rad52*Δ homozygotes (Figures 2 and 4). Perhaps, the much less dramatic effect of the loss of Rad52 in the presence of Rad51 reflects the ability of Rad59, which also possesses single-stranded DNA annealing activity to act like Rad52, a notion supported by the synergistically reduced frequencies of T2 observed previously in *rad52*Δ*/rad52*Δ *rad59*Δ*/rad59*Δ double homozygotes [27], [56], [57], [58]. However, experiments suggest that Rad51 can only form nucleoprotein filaments when Rad52 is present to displace RPA from single-stranded DNA, and Rad59 can neither displace RPA from single-stranded DNA, nor anneal single-stranded DNA molecules bound by RPA [9], [56], [57], [85], [86], [87]. This suggests that the contributions of Rad51 and Rad59 to T2 in the *rad52*Δ*/rad52*Δ homozygotes may not involve their functions in filament formation or annealing, While the role of Rad59 could possibly be related to its participation in the removal of non-homologous tails, the fact that loss of filament formation conferred by *rad51*Δ and *rad52-329* both suppress the necessity for Rad59 counters that suggestion as the complete absence of Rad52 conferred by *rad52*Δ should also block filament formation (Figures 2 and 4). The role of Rad51 in translocation formation by SSA clearly merits further investigation.

While the role of Rad51 filaments in T2 is unknown, the genetic data described here, and previously support the following speculative model (Table 1, Figures 1–4)[27], [36], [49]. Following the creation of Rad51 filaments at DSBs, Srs2 removes Rad51, permitting the complementary sequences on 3′ single strands to be annealed by Rad52 and Rad59. Rad59 then collaborates with Msh2-Msh3 and Rad1-Rad10 to coordinate the removal of the non-homologous tails created by annealing the complementary segments of the 3′ single strands. Epistasis analysis suggests that Srs2 may also play a role in removing Rad51 that remains associated with non-homologous tails, or has reassociated, which may inhibit their removal by Rad1-Rad10 (Figure 3). Removal of the tails facilitates repair synthesis and ligation, forming a covalent joint and a translocation chromosome. In contrast, when Rad51 filaments do not form at DSBs, Rad52 anneals complementary single strands without the assistance of Srs2 and Rad59. Without Rad59 specifying the use of Msh2-Msh3 and Rad1-Rad10 for the removal of non-homologous tails, other factors execute this task so that repair synthesis and ligation can complete the recombination event [49]. All aspects of this model are currently under investigation at the genetic and molecular levels.

Because all eukaryotes likely share with budding yeast a genomic structure that features an abundance of short repetitive sequences, non-conservative recombination between unlinked repeats must be minimized in favor of conservative recombination so that genome stability can be maintained. Also conserved from yeast to man is the central strand exchange protein, Rad51, which facilitates conservative HR, even between non-allelic sequences (Table 1, Figure 2)[12], [17], [88], [89], [90], [91], [92], [93], [94], [95], [96]. While Rad52 is also conserved in most eukaryotes, its function in facilitating nucleoprotein filament formation by Rad51 appears to have been replaced in many species by Brca2 [23], [46], [47], [97], [98], [99], [100], [101], [102], [103], [104], [105]. Fascinatingly, like the *rad51*Δ and *rad52-329* mutations in yeast, mutations that disable Rad51, or disrupt the interaction between Brca2 and Rad51 in worms, flies and mammals block conservative GC and stimulate non-conservative SSA [17], [23], [43], [44], [45]. Given the parallels with yeast, it is tempting to speculate that defects in filament formation in higher eukaryotes release the restriction against a mechanism of SSA that is analogous to the one described here. Given the probable link between SSA and the non-allelic HR observed in tumors and during genome evolution, revealing the molecular basis of this mechanism could have far reaching implications [32], [33], [106], [107].

## Materials and Methods

### Yeast strain and plasmid construction

All yeast strains used in this study were isogenic, and were grown, maintained and manipulated using standard techniques (Table S2)[108]. Plasmids were constructed using standard molecular biological techniques [109]. Construction of the gene conversion and translocation substrates was described previously [27], [49], [110]. Construction of the *rad1*Δ [111], *rad51*Δ [112], *rad52*Δ [113], *rad59*Δ [27], and *his3*Δ*200* alleles was described previously [114]. All strains in which HO-mediated DSBs were generated at recombination substrates were homozygous for *MATa::LEU2*, an allele that disrupts the mating type locus, and prevents the creation of DSBs.

The *rad52-329* allele used in this study was identical to the allele that was described previously and was constructed as follows (Tsukamoto 2003): A DNA fragment containing a *Bam* HI site juxtaposed to the *RAD52* stop codon and approximately 850 bp of DNA downstream from the *RAD52* locus was amplified from yeast genomic DNA by PCR using primers P1 (5′-CGG GAT CCC TGA AAC GCT TCC TGG CCG-3′ and P2-(5′-GGG TCG ACG TCC AAG AAA TAC ATT GG-3′). The resulting fragment was cloned into the *Sma* I site of pBlueScript to generate pBS-rad52-329. An 850 bp fragment carrying the *RAD52* stop codon and downstream DNA was excised from pBS-rad52-329 with *Bam* HI, and inserted into the *Bam* HI site of the plasmid YIp356R-RAD52, which was constructed by inserting a 1.9 kb *Nhe* I fragment carrying 1 kb of DNA flanking the 5′ end of the *RAD52* gene and the first 900 bp of the *RAD52* coding sequence into the *Xba* I site of YIp356R [115]. This created YIp356R-rad52-329, which contains DNA from both sides of the *RAD52* locus flanking *rad52-329*, an allele of *RAD52* missing the DNA encoding the final 178 amino acids of the coding sequence. YIp356R-rad52-329 was linearized with *Bgl* II to target its insertion into the *RAD52* locus, and used to transform yeast to uracil prototrophy. Uracil prototrophic transformants were grown in the presence of 5-fluoroorotic acid to select for Ura^-^ recombinants that were screened by PCR and Southern blotting to ascertain which clones contained the *rad52*-*329* allele at the *RAD52* locus (M. Navarro and A. Bailis, unpublished results)[116].

The *srs2*Δ allele used in this study was generated as follows: A 3,145 bp fragment of DNA corresponding to the 537 bp of genomic sequence flanking the 5′ end of the *SRS2* coding sequence, and the first 2,608 bp of the *SRS2* coding sequence was amplified from genomic DNA by PCR using the primers 5′SRS2 (5′ GCT CAC GAT CTA CGA GAT GCG GC-3′) and 3′SRS2 (5′-GCC ATT GAT TTT GGA TGG GCG-3′). This fragment was cloned into the *Sma* I site of pBlueScript, creating pLAY482. pLAY482 was linearized in the *SRS2* coding sequence by digestion with *Msc* I, and the 1.7 kb *Pvu* II *TRP1* fragment from pUC-TRP inserted to create pLAY484. pLAY484 was digested with *Spe* I and *Xho* I to liberate the *srs2::TRP1* disruption cassette that was used to transform yeast to prototrophy for tryptophan. The resulting tryptophan prototrophs were screened by Southern blot analysis to confirm the presence of the disrupted allele at the *SRS2* locus (G. Manthey and A. Bailis, unpublished results).

### Ectopic gene conversion (EGC) frequencies

The design and execution of the DSB-stimulated *SAM* ectopic gene conversion assay in diploid strains was described in detail previously [49], [117]. Briefly, recombination was initiated by cutting at the HO cut-site in *sam1-*Δ*Bgl II-HOcs* at the *SAM1* locus on copy of chromosome XII by HO endonuclease expressed from a galactose-inducible *HO* gene integrated into the *TRP1* locus on one copy of chromosome IV. Cutting *sam1-*Δ*Bgl II-HOcs* with HO creates broken ends that share 279 bp and 1.4 kb of homology with the *sam1-*Δ*Sal I* donor sequence inserted into the *HIS3* locus on one copy of chromosome XV. The *sam1-*Δ*Bgl II-HOcs* and *sam1-*Δ*Sal I* substrates have opposite orientations relative to their centromeres, preventing the isolation of reciprocal recombinants. For each strain, 10, one or five ml cultures of complete synthetic medium containing 3% glycerol and 3% lactate as carbon sources and supplemented with 100 µg/ml S-adenosyllmethionine (AdoMet) were inoculated with single colonies and grown overnight, or two days at 30°C before the addition of 0.1 ml, or 0.5 ml aliquots of 20% galactose and a further four hour incubation at 30°C. Appropriate dilutions of each galactose-induced culture were plated on to YPD (2% dextrose, 2% bacto-peptone, 1% yeast extract) agar supplemented with 100 µg/ml AdoMet and incubated at 30°C for four to five days to determine viability. Appropriate volumes of the galactose-induced cultures were also plated on to unsupplemented YPD agar and incubated at 30°C for four to five days to determine the number of AdoMet prototrophic recombinants. The number of AdoMet prototrophic recombinants was divided by the number of viable cells plated to determine the frequency of EGC. Median EGC frequencies from a least 10 independent cultures were determined for each genotype, and 95% confidence intervals were determined using a table [117]. P values were determined using the Mann-Whitney test with the Prism (Graphpad) software package.

### Spontaneous translocation rates

Rates of spontaneous translocation in wild type and mutant diploid strains were determined as previously described [27]. For each genotype a minimum of ten 10 ml YPD cultures were inoculated with single colonies and grown overnight to two days at 30°C. Appropriate dilutions of the cultures were plated on to YPD agar and incubated at 30°C for three to four days to determine viability. Appropriate volumes of culture were plated on to synthetic medium lacking histidine and incubated at 30°C for three to four days to assess translocation formation by HR between the *his3-*Δ*3*′ and *his3-*Δ*5*′ substrates (Figure 1). Rates were determined by the method of the median and 95% confidence intervals were determined using a table [117], [118]. In the *rad52*Δ*/rad52*Δ homozygotes, where over 90% of the trials resulted in no recombinants, a theoretical rate was calculated using fluctuation analysis based upon 10% recovery of histidine prototrophic recombinants with the actual rate expected to be at or below this number [119]. Selected His^+^ recombinants were examined by genomic Southern and chromosome blot analyses to determine the nature of the recombination event as described previously (G. Manthey and A. Bailis, unpublished results)[27].

### DSB-stimulated translocation frequencies

Frequencies of translocation formation in wild type and mutant diploid strains subsequent to DSB formation via HO-endonuclease cleavage were determined as described previously with slight modifications [27]. Briefly, one ml cultures of YP-Gly/Lac (2% yeast extract, 2% bacto-peptone, 3% glycerol, 3% lactic acid, pH 5.8) were inoculated with single colonies and incubated 16–24 hours at 30°C. Galactose was added to a final concentration of 2% to induce the expression of the HO-endonuclease. Southern blot analysis confirmed complete cleavage of the substrates following the addition of galactose (G. Manthey and A. Bailis, unpublished data). After four hours of induction appropriate dilutions of the culture were plated to YPD to determine cell viability and to medium lacking histidine to determining the number of histidine prototrophs. The frequency of translocation was determined by dividing the number of histidine prototrophs by the number of viable cells in the culture. Median translocation frequencies from a least 10 independent cultures were reported and the 95% confidence intervals determined using a table [117]. P values were determined using the Mann-Whitney test with the Prism (Graphpad) software package. Selected His^+^ recombinants were subjected to genomic Southern and chromosome blot analyses as described previously (G. Manthey and A. Bailis, unpublished results)[27].

### Plating efficiencies

Plating efficiencies were determined as described previously [27]. Cells containing the translocation substrates were cultured in YP-Gly/Lac medium overnight, cell counts made by hemacytometer, appropriate numbers of cells plated on to YPD agar, and the plates incubated at 30°C for three to four days. Following the addition of galactose to a final concentration of 2% and four hours of additional incubation at 30°C, cells were again counted by hemacytometer, appropriate numbers of cells plated on to YPD agar, and plates incubated at 30° for three to four days. Plating efficiency was calculated by dividing the number of colonies appearing on the YPD plates by the number of cell bodies plated and multiplying the quotient by 100. The median plating efficiencies from at least 10 independent trials were reported and the 95% confidence intervals determined from a table [117]. P values were determined using the Mann-Whitney test with the Prism (Graphpad) software package.

### Contour-clamped homogenous electric field (CHEF) analysis

Chromosomes from selected His^+^ recombinant colonies were prepared in agarose plugs using an established protocol [120]. Chromosomes were separated on 1% agarose gels with a Bio-Rad CHEF-DR II apparatus (BioRad, Hercules, CA) using parameters that were described previously [27]. The separated chromosomes were visualized after staining with 1 µg/ml ethidium bromide for 30 min and photographed. Ethidium stained chromosomes were irradiated with 60 mJoules of UV in a Stratagene Stratalinker 1800 to nick the DNA, and destained for 30 min in deionized, distilled water. The chromosomes were transferred from the gel to a nylon membrane (Hybond N^ = ^, GE Healthcare, Waukesha, WI) by electroblotting with a Genie Blotter apparatus (Idea Scientific Co., Minneapolis, MN). These were probed with a 1.8 kb *Bam* HI *HIS3* genomic clone corresponding to sequences from 469 bp upstream of the *HIS3* open reading frame to 634 bp downstream that had been labeled with ^32^P by random priming using a Megaprime DNA labeling kit (GE Healthcare). Blots were exposed to film, and the film developed using a Konica Minolta SRX-101A processor (Konica Minolta USA, Ramsey, NJ).

## Supporting Information

Table S1 T2 frequencies in wild-type and mutant diploid strains. Median frequencies are displayed. 95% confidence intervals are in parentheses. Fold differences from wild-type are in brackets.
(0.05 MB PDF)

Table S2 *Saccharomyces cerevisiae* strains used in this study. All used in this study were isogenic. Full genotypes available upon request.
(0.07 MB PDF)

Table S3 Plating efficiencies (PE) “Pre” and “Post” HO endonuclease cutting at *his3-Δ5′* and *his3-Δ3′* were determined as described in the Materials and Methods. Median frequencies are displayed. 95% confidence intervals are displayed parentheses. Fold differences from wild type are indicated in brackets.
(0.04 MB PDF)
