# Supplementary material for: Rad51 Inhibits Translocation Formation by Non-Conservative Homologous Recombination in Saccharomyces cerevisiae
Source: PLoS One. 2010 Jul 29;5(7):e11889. doi: 10.1371/journal.pone.0011889 (PMC2912366; doi:10.1371/journal.pone.0011889)
Supplement: Table S1 — T2 frequencies in wild-type and mutant diploid strains. Median frequencies are displayed. 95% confidence intervals are in parentheses. Fold differences from wild-type are in brackets. (0.05 MB PDF) [file pone.0011889.s001.pdf]

Table S1. T2 Frequencies in wild-type and mutant diploid strains.

Genotype	60 bp	300 bp
Wild-type	$6.8x10^{-3}$ (2.5, 8.0)	$2.2x10^{-2}$ (1.4, 3.1)
rad51∆/rad51∆	$2.4x10^{-2}$ (1.9, 4.0) [+3.6]	$6.0x10^{-2}$ (3.8, 12) [+2.7]
rad52∆/rad52∆	$6.0x10^{-4}$ (2.5, 9.8) [-11.3]	$3.0x10^{-3}$ (1.6, 4.3) [-7.3]
rad52-329/rad52-329	$1.1x10^{-1}$ (0.9, 1.2) [+16.2]	$2.0x10^{-2}$ (1.4, 3.1) [-1.1]
rad59∆/rad59∆	$8.3x10^{-5}$ (6.8, 10) [-82.0]	$5.3x10^{-4}$ (4.0, 6.6) [-41.5]
rad1∆/rad1∆	$9.1x10^{-5}$ (5.4, 17) [-74.7]	$1.9x10^{-4}$ (1.3, 4.6) [-115.8]
srs2∆/srs2∆	$8.6x10^{-5}$ (6.3, 11) [-79.1]	$8.0x10^{-4}$ (7.5, 11) [-27.5]
rad52-329/rad52-329 rad51∆/rad51∆	$5.4x10^{-2}$ (3.4, 13) [+7.9]	$1.2x10^{-1}$ (0.8, 2.5) [+5.5}
rad52-329/rad52-329 rad59∆/rad59∆	$1.0x10^{-2}$ (0.8, 1.9) [+1.5]	$3.5x10^{-2}$ (1.8, 5.0) [+1.6]
rad52-329/rad52-329 rad1∆/rad1∆	$4.0x10^{-3}$ (2.6, 6.3) [-1.7]	$2.3x10^{-2}$ (1.5, 3.1) [+1.1]
rad51∆/rad51∆ rad59∆/rad59∆	$2.7x10^{-2}$ (1.5, 5.2) [+4.0]	$4.7x10^{-2}$ (3.5, 11) [+2.1]
rad51∆/rad51∆ rad1∆/rad1∆	$8.0x10^{-4}$ (4.7, 14) [-8.5]	$2.2x10^{-2}$ (3.5, 11) [+1.0]
rad51∆/rad51∆ rad52∆/rad52∆	$3.0x10^{-5}$ (1.2, 5.5) [-226.7]	$4.9x10^{-5}$ (4.1, 10) [-449.0]
rad52∆/rad52∆ srs2∆/srs2∆	$6.1x10^{-5}$ (3.7, 19) [-111.5]	$3.3x10^{-4}$ (1.6, 13) [-66.7]
rad52-329/rad52-329 srs2∆/srs2∆	$1.3x10^{-1}$ (0.9, 1.9) [+19.1]	$2.1x10^{-1}$ (1.6, 2.9) [+9.6]
srs2∆/srs2∆ rad59∆/rad59∆	$1.2x10^{-5}$ (0.5, 2.0) [-566.7]	$1.8x10^{-4}$ (1.5, 2.5) [-122.2]
srs2∆/srs2∆ rad1∆/rad1∆	$1.0x10^{-5}$ (0.4, 2.5) [-680.0]	$2.3x10^{-4}$ (1.8, 4.0) [-95.7]
rad52-329/rad52-329 srs2∆/srs2∆ rad59∆/rad59∆	$2.4x10^{-3}$ (1.1, 3.4) [-2.8]	$3.6x10^{-2}$(3.4, 6.0) [+1.6]
rad52-329/rad52-329 srs2∆/srs2∆ rad1∆/rad1∆	$1.8x10^{-3}$ (0.7, 6.0) [-3.8]	$4.9x10^{-2}$ (3.4, 6.0) [+2.2]
