# Supplementary material for: Rad51 Inhibits Translocation Formation by Non-Conservative Homologous Recombination in Saccharomyces cerevisiae
Source: PLoS One. 2010 Jul 29;5(7):e11889. doi: 10.1371/journal.pone.0011889 (PMC2912366; doi:10.1371/journal.pone.0011889)
Supplement: Table S2 — Saccharomyces cerevisiae strains used in this study. All used in this study were isogenic. Full genotypes available upon request. (0.07 MB PDF) [file pone.0011889.s002.pdf]

Table S2. *Saccharomyces cerevisiae* strains used in this study.

Strain	Relevant Genotype	Reference
ABX2652	*MATa::LEU2/MATa::LEU2, his3-11, 15/HIS3-sam1*Δ*SalI, sam1*Δ*BglII-HOcs/sam1::LEU2, sam2::HIS3/sam2::HIS3, trp1-1/trp1::GAL-HO-KanMX*	Pannunzio *et al.* 2010
ABM264	Same as ABX2652 except *rad52::TRP1/rad52::TRP1*	Pannunzio *et al.* 2010
ABM269	Same as ABX2652 except *rad52-329/rad52-329*	This study
ABM272	Same as ABX2652 except *rad51::LEU2/rad51::LEU2*	This study
ABM147	*MAT***a**/α, *his3-*Δ*200/his3-*Δ*3'-HOcs, leu2-3,112/ leu2::HOcs-his3*Δ*5'*$_{(300)}$	Pannunzio *et al.* 2008
ABM153	Same as ABM147 except *rad51::LEU2/rad51::LEU2*	Pannunzio *et al.* 2008
ABX1691	Same as ABM147 except *rad52::TRP1/rad52::TRP1*	Pannunzio *et al.* 2008
ABM216	*MATa::LEU2/MATa::LEU2, his3-*Δ*200/his3-*Δ*3'-HOcs*$_{(MUT)}$, *leu2-3,112/leu2::HOcs-his3*Δ*5'*$_{(300)}$	This study
ABX213	Same as ABM216 except *rad52-329/rad52-329*	This study
ABM227	Same as ABM216 except *rad52::TRP1/rad52::TRP1*	This study
ABM234	Same as ABM216except *rad51::LEU2/rad51::LEU2*	This study
ABM198	*MATa::LEU2/MATa::LEU2, his3-*Δ*200/his3-*Δ*3'-HOcs, leu2-3,112/leu2::HOcs-his3*Δ*5'*$_{(60)}$, *trp1-1/trp1::GAL-HO-KanMX*	This study
ABM202	Same as ABM198 except *rad52-329/rad52-329*	This study
ABM207	Same as ABM198 except *rad59::LEU2/rad59::LEU2*	This study
ABM221	Same as ABM198 except *rad52-329/rad52-329, rad1::LEU2/rad1::LEU2*	This study
ABM226	Same as ABM198 except *rad52-329/rad52-329, rad59::LEU2/rad59::LEU2*	This study
ABM230	Same as ABM198 except *rad52::TRP1/rad52::TRP1*	This study
ABM231	Same as ABM198 except *rad51::LEU2/rad51::LEU2*	This study
ABM236	Same as ABM198 except *rad51::LEU2/rad51::LEU2, rad52-329/rad52-329*	This study
ABM237	Same as ABM198 except *rad1::LEU2/rad1::LEU2*	This study
ABM254	Same as ABM198 except *rad51::LEU2/rad51::LEU2, rad1::LEU2/rad1::LEU2*	This study
ABM261	Same as ABM198 except *rad51::LEU2/rad51::LEU2, rad59::LEU2/rad59::LEU2*	This study
ABM283	Same as ABM198 except *srs2::TRP1/srs2::TRP1, rad1::LEU2/rad1::LEU2*	This study
ABM284	Same as ABM198 except *srs2::TRP1/srs2::TRP1*	This study
ABM285	Same as ABM198 except *rad52-329/rad52-329, srs2::TRP1/srs2::TRP1, rad1::LEU2/rad1::LEU2*	This study
ABM291	Same as ABM198 except *rad52-329/rad52-329, srs2::TRP1/srs2::TRP1*	This study
ABM311	Same as ABM198 except *srs2::TRP1/srs2::TRP1, rad59::LEU2/rad59::LEU2*	This study
ABM312	Same as ABM198 except *rad52-329/rad52-329, srs2::TRP1/srs2::TRP1, rad59::LEU2/rad59::LEU2*	This study
ABM314	Same as ABM198 except *rad51::LEU2/rad51::LEU2,*	This study

	rad52::TRP1/rad52::TRP1	
ABM322	Same as ABM198 except *rad52::TRP1/rad52::TRP1, srs2::TRP1/srs2::TRP1*	This study
ABM197	*MATa::LEU2/MATa::LEU2, his3-Δ200/his3-Δ3'-HOcs, leu2-3,112/leu2::HOcs-his3Δ5'$_{(300)}$, trp1-1/trp1::GAL-HO-KanMX*	This study
ABM201	Same as ABM197 except *rad52-329/rad52-329*	This study
ABM208	Same as ABM197 except *rad59::LEU2/rad59::LEU2*	This study
ABM222	Same as ABM197 except *rad52-329/rad52-329, rad1::LEU2/rad1::LEU2*	This study
ABM225	Same as ABM197 except *rad52-329/rad52-329, rad59::LEU2/rad59::LEU2*	This study
ABM232	Same as ABM197 except *rad51::LEU2/rad51::LEU2*	This study
ABM235	Same as ABM197 except *rad51::LEU2/rad51::LEU2, rad52-329/rad52-329*	This study
ABM238	Same as ABM197 except *rad1::LEU2/rad1::LEU2*	This study
ABM250	Same as ABM197 except *rad51::LEU2/rad51::LEU2, rad59::LEU2/rad59::LEU2*	This study
ABM253	Same as ABM197 except *rad51::LEU2/rad51::LEU2, rad1::LEU2/rad1::LEU2*	This study
ABM286	Same as ABM197 except *srs2::TRP1/srs2::TRP1*	This study
ABM287	Same as ABM197 except *srs2::TRP1/srs2::TRP1, rad1::LEU2/rad1::LEU2*	This study
ABM290	Same as ABM197 except *rad52-329/rad52-329, srs2::TRP1/srs2::TRP1, rad1::LEU2/rad1::LEU2*	This study
ABM292	Same as ABM197 except *rad52-329/rad52-329, srs2::TRP1/srs2::TRP1*	This study
ABM293	Same as ABM197 except *srs2::TRP1/srs2::TRP1, rad59::LEU2/rad59::LEU2*	This study
ABM315	Same as ABM197 except *rad51::LEU2/rad51::LEU2, rad52::TRP1/rad52::TRP1*	This study
ABM319	Same as ABM197 except *rad52-329/rad52-329, srs2::TRP1/srs2::TRP1, rad59::LEU2/rad59::LEU2*	This study
ABM321	Same as ABM197 except *rad52::TRP1/rad52::TRP1, srs2::TRP1/srs2::TRP1*	This study
