# Supplementary material for: Rad51 Inhibits Translocation Formation by Non-Conservative Homologous Recombination in Saccharomyces cerevisiae
Source: PLoS One. 2010 Jul 29;5(7):e11889. doi: 10.1371/journal.pone.0011889 (PMC2912366; doi:10.1371/journal.pone.0011889)
Supplement: Table S3 — Plating efficiencies (PE) “Pre” and “Post” HO endonuclease cutting at his3-Δ5′ and his3-Δ3′ were determined as described in the Materials and Methods. Median frequencies are displayed. 95% confidence intervals are displayed parentheses. Fold differences from wild type are indicated in brackets. (0.04 MB PDF) [file pone.0011889.s003.pdf]

**Table S3.** Plating efficiencies in wild type and homozygous mutant diploid strains.

| Genotype | Plating efficiency | |
|---|---|---|
| | Pre-induction | Post-induction |
| Wild type | $2.1 \times 10^{-1}$ | $1.6 \times 10^{-1}$ |
| | (1.4, 3.1) | (1.1, 3.6) |
| *srs2Δ/srs2Δ* | $3.7 \times 10^{-1}$ | $2.6 \times 10^{-1}$ |
| | (3.3, 5.3) | (2.1, 3.3) |
| | [+1.8] | [+1.6] |
| *srs2Δ/srs2Δ rad1Δ/rad1Δ* | $4.3 \times 10^{-1}$ | $2.4 \times 10^{-1}$ |
| | (3.9, 5.1) | (1.4, 3.7) |
| | [+2.1] | [+1.5] |
| *srs2Δ/srs2Δ rad59Δ/rad59Δ* | $2.6 \times 10^{-1}$ | $2.0 \times 10^{-1}$ |
| | (2.2, 4.5) | (1.6, 2.9) |
| | [+1.2] | [+1.3] |
